# A Computational Synaptic Antibody Characterization Tool for Array Tomography

**DOI:** 10.3389/fnana.2018.00051

**Published:** 2018-07-17

**Authors:** Anish K. Simhal, Belvin Gong, James S. Trimmer, Richard J. Weinberg, Stephen J Smith, Guillermo Sapiro, Kristina D. Micheva

**Affiliations:** ^1^Electrical and Computer Engineering, Duke University, Durham, NC, United States; ^2^Department of Neurobiology, Physiology and Behavior, University of California, Davis, Davis, CA, United States; ^3^Department of Cell Biology and Physiology, University of North Carolina, Chapel Hill, NC, United States; ^4^Synapse Biology, Allen Institute for Brain Science, Seattle, WA, United States; ^5^Department of Biomedical Engineering, Department of Computer Science, Department of Mathematics, Duke University, Durham, NC, United States; ^6^Molecular and Cellular Physiology, School of Medicine, Stanford University, Stanford, CA, United States

**Keywords:** synapse, antibodies, array tomography, synapse detection, proteometric composition, automatic algorithms, antibody characterization

## Abstract

Application-specific validation of antibodies is a critical prerequisite for their successful use. Here we introduce an automated framework for characterization and screening of antibodies against synaptic molecules for high-resolution immunofluorescence array tomography (AT). The proposed Synaptic Antibody Characterization Tool (SACT) is designed to provide an automatic, robust, flexible, and efficient tool for antibody characterization at scale. SACT automatically detects puncta of immunofluorescence labeling from candidate antibodies and determines whether a punctum belongs to a synapse. The molecular composition and size of the target synapses expected to contain the antigen is determined by the user, based on biological knowledge. Operationally, the presence of a synapse is defined by the colocalization or adjacency of the candidate antibody punctum to one or more reference antibody puncta. The outputs of SACT are automatically computed measurements such as target synapse density and target specificity ratio that reflect the sensitivity and specificity of immunolabeling with a given candidate antibody. These measurements provide an objective way to characterize and compare the performance of different antibodies against the same target, and can be used to objectively select the antibodies best suited for AT and potentially for other immunolabeling applications.

## 1. Introduction

Antibodies are an indispensable tool for the modern biologist. Their high-affinity binding to specific target molecules makes it possible to detect, isolate, and manipulate the function of these molecules. A staggering number of antibodies are available to the research community, as are many options to make new antibodies. However, since antibodies are biological tools employed in complex systems, they can be very difficult to evaluate and to use in a predictable and reproducible way. A large volume of misleading data has been published based on results from antibodies that did not perform as assumed (Anderson and Grant, 2006; Rhodes and Trimmer, 2006; Baker, 2015). Recognizing this problem, there has been substantial progress in optimizing antibody production and validation (Nilsson et al., 2005; Gong et al., 2016). The importance of establishing reliable practices for antibody use is now widely accepted, and many companies are adopting transparent practices for rigorous antibody validation (Fritschy et al., 1998; Uhlen et al., 2016). The performance of antibodies, however, is application-specific (Lorincz and Nusser, 2008), and the reliable performance of an antibody in one application does not guarantee its suitability for another application. For example, an antibody that yields a single band on an immunoblot analysis of a tissue homogenate may prove wholly unsatisfactory for immunohistochemistry on sections of fixed tissue. Moreover, the same antibody that yields a robust and specific signal in immunohistochemical labeling of tissue sections prepared under one set of conditions may yield a weak or noisy signal on comparable samples prepared under different conditions (Fritschy et al., 1998; Fukaya and Watanabe, 2000). Therefore, it is up to the individual user to validate antibodies for other applications and conditions. This task is especially crucial for applications whose chemistry differs substantially from standard immunoblots.

Array tomography (AT) is a technique that involves immunolabeling and imaging of serial arrays of ultrathin (~70 nm) plastic-embedded tissue slices of aldehyde-fixed tissue (Micheva and Smith, 2007; Micheva et al., 2010). While embedding tissue in resin has multiple advantages, the embedding process requires tissue dehydration, infiltration in plastic resin, and subsequent resin polymerization, all of which can modify the protein structure and chemical state and thus have a major impact on its immunoreactivity. Identifying antibodies that yield robust and specific immunolabeling of target proteins in plastic sections is a daunting task. AT is a high-resolution/high-throughput tool well-suited for the study of synapses in the mammalian brain; unfortunately, finding antibodies that selectively label synapses presents additional challenges, due to their small size, high density, and overall neurochemical complexity and diversity.

**Figure 1:**
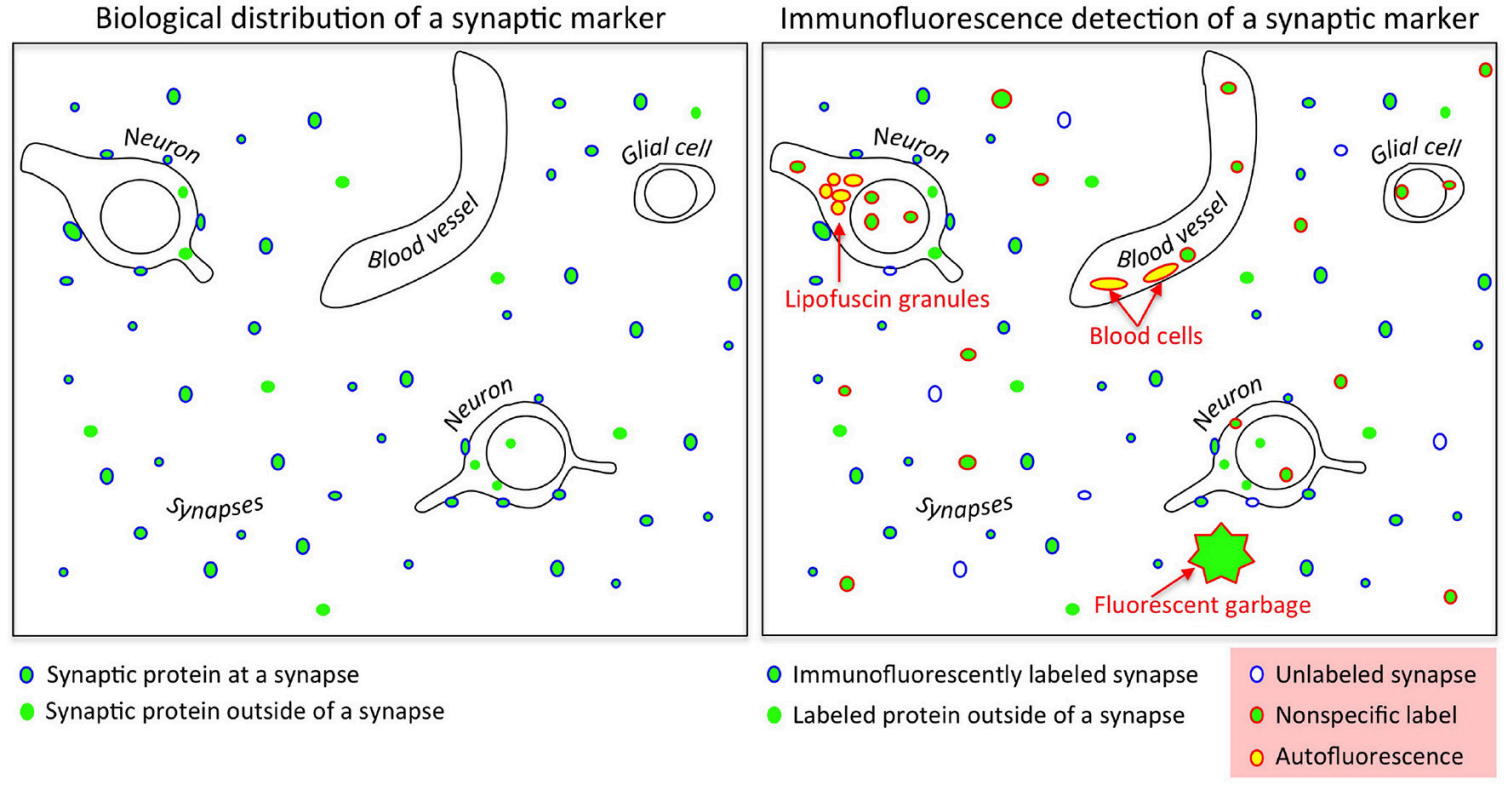
Challenges in evaluating synaptic antibodies. **Left**: Synaptic proteins are found not only at synapses, but also outside of synapses at sites of synthesis and transport; many synaptic proteins also lie in other subcellular compartments reflecting other functions (such as transcriptional regulators). **Right**: Immunofluorescence detection of synaptic proteins is confounded by nonspecific binding of antibodies, low efficiency of target protein detection, fluorescent contaminants such as dust particles, and tissue autofluorescence (e.g., lipofuscin granules and blood cells).

The primary criterion for evaluating antibody performance for immunohistochemistry is determining whether the labeling pattern is consistent with the known tissue characteristics of its target protein. For an antibody against a synaptic protein, the immunolabeling must be localized at synapses. Though conceptually straightforward, the practical evaluation of this criterion is difficult and often involves a number of subjective and time-consuming decisions. A synapse can be unambiguously identified via electron microscopy, but this approach is too time-consuming and expensive to be practical for large scale (~ 100 antibodies against a target protein) antibody screening tests. A more efficient strategy is to double label the same sample with another antibody already known to localize at synapses, and measure colocalization (Micheva et al., 2010; Weiler et al., 2014). While effective, this method presents a number of challenges. Synaptic proteins are typically expressed in high concentrations at synapses; however, these proteins are also present at other subcellular locations. Furthermore, synapses display a high level of protein diversity, so many synapses may completely lack a particular synaptic protein. Adding to the uncertainty, other sources of fluorescence can confuse the interpretation of the images. These sources of “noise” include signal arising from autofluorescent tissue constituents such as lipofuscin granules, blood cells, contamination, and defects such as tissue folds created during sample processing (Figure 1). The trained eye of an expert can usually discern the different fluorescence sources and pick out the specific immunolabel, but this is a subjective and non-quantitative assessment. Furthermore, it can be extremely time consuming, especially when examining a large number of antibodies against the same antigen, as may be required during antibody production (Gong et al., 2016).

Accordingly, there is an urgent need for an efficient and robust framework for evaluation of synaptic antibodies. Here, we introduce the Synaptic Antibody Characterization Tool (SACT), which provides automatic and quantitative measurements of the intensity and specificity of immunolabel and enables the objective characterization and comparison of multiple synaptic antibodies for AT at scale. Because the terms used in this paper are specific to the domain, see the ‘Definitions’ box for further explanation.

**Definitions**

**Candidate Antibody**—Antibody being tested for the antigen of interest.

**Reference Antibody**—A previously validated antibody for an antigen known to colocalize with or lie adjacent to the candidate antibody's antigen of interest.

**Colocalization**—When two or more antibody puncta occupy the same physical space, as shown in Box B. This is often the case when both antibodies have presynaptic targets, or both have postsynaptic targets.

**Adjacency**—When two or more antibody puncta are physically next to each other in 3D space, as shown in Box A. This may be the case when one antibody has a presynaptic target and the other a postsynaptic target.

**Punctum**—A small blob of signal defined in 2D or 3D space resulting from imaging an antibody applied on a tissue. In Box A, one punctum is the green circle, another is the blue triangle.

**Target synapse**—A synapse that contains the candidate antibody target, based on biological knowledge. Operationally, the presence of a synapse is defined by the adjacency (Box A) or colocalization (Box B) of the candidate antibody punctum to one or more reference antibody puncta. Box C is an example where the two puncta are neither adjacent nor colocalize, and thus do not form a synapse.

**Target specificity ratio**—The proportion of candidate antibody puncta that lie at target synapses.

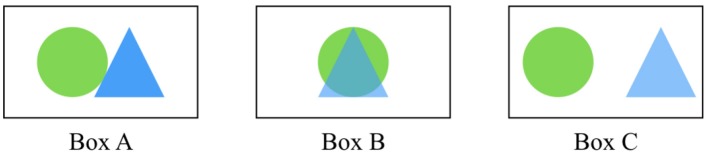

*The circle and triangle represent different imaged antibody puncta detected by two different antibodies*.

## 2. Methods

### 2.1. Overview

**Figure 2:**
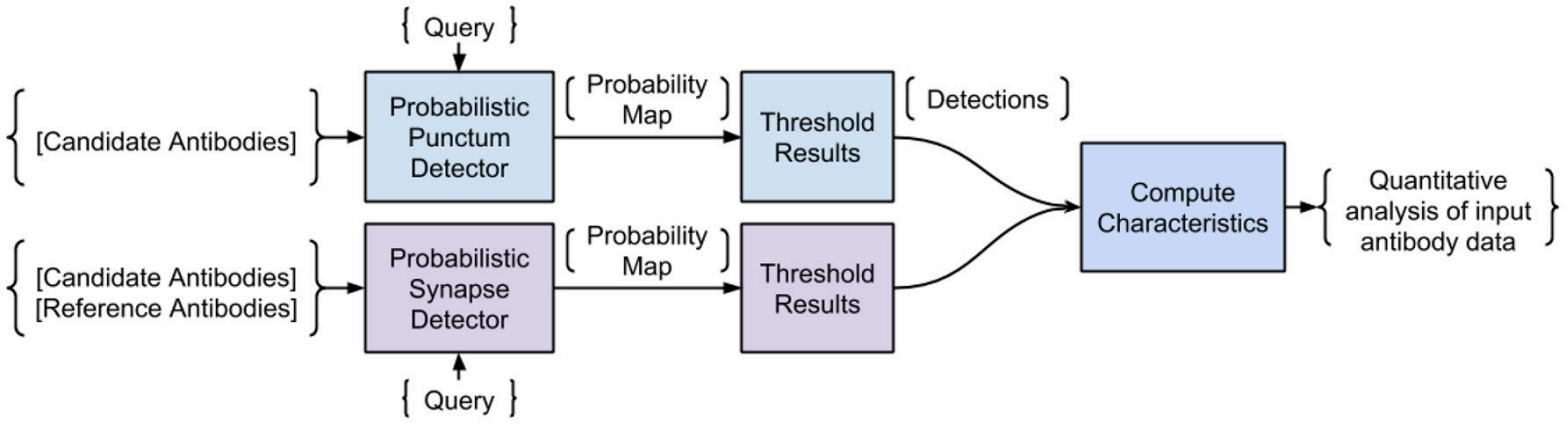
Pipeline of the Synaptic Antibody Characterization Tool (SACT). SACT combines a probabilistic punctum detector (top row) with a probabilistic synapse detector (Simhal et al., 2017) to determine the properties of the candidate antibody.

The proposed Synaptic Antibody Characterization Tool (SACT) was developed for the quantitative assessment of antibodies against synaptic targets used for AT. It automatically detects puncta of immunofluorescence labeling from candidate antibodies and computes their density, size and size variability. SACT then determines whether a punctum belongs to a target synapse or not, by using the previously described probabilistic synapse detector (Simhal et al., 2017). The presence of a target synapse is defined by the colocalization or adjacency of the candidate antibody punctum to one or more reference antibody puncta. This allows the output of two additional measures: density of target synapses, that is the synapses containing candidate antibody puncta, and target specificity ratio, which is the fraction of candidate antibody puncta that are at the target synapses. These measurements provide an objective way to characterize the sensitivity and specificity of a candidate antibody and to compare its performance to other antibodies. The approach is outlined in Figure 2. We should note that SACT is a framework; additional measurements can be added and adapted as needed depending on the desired antibody characterization features.

**Figure 3:**
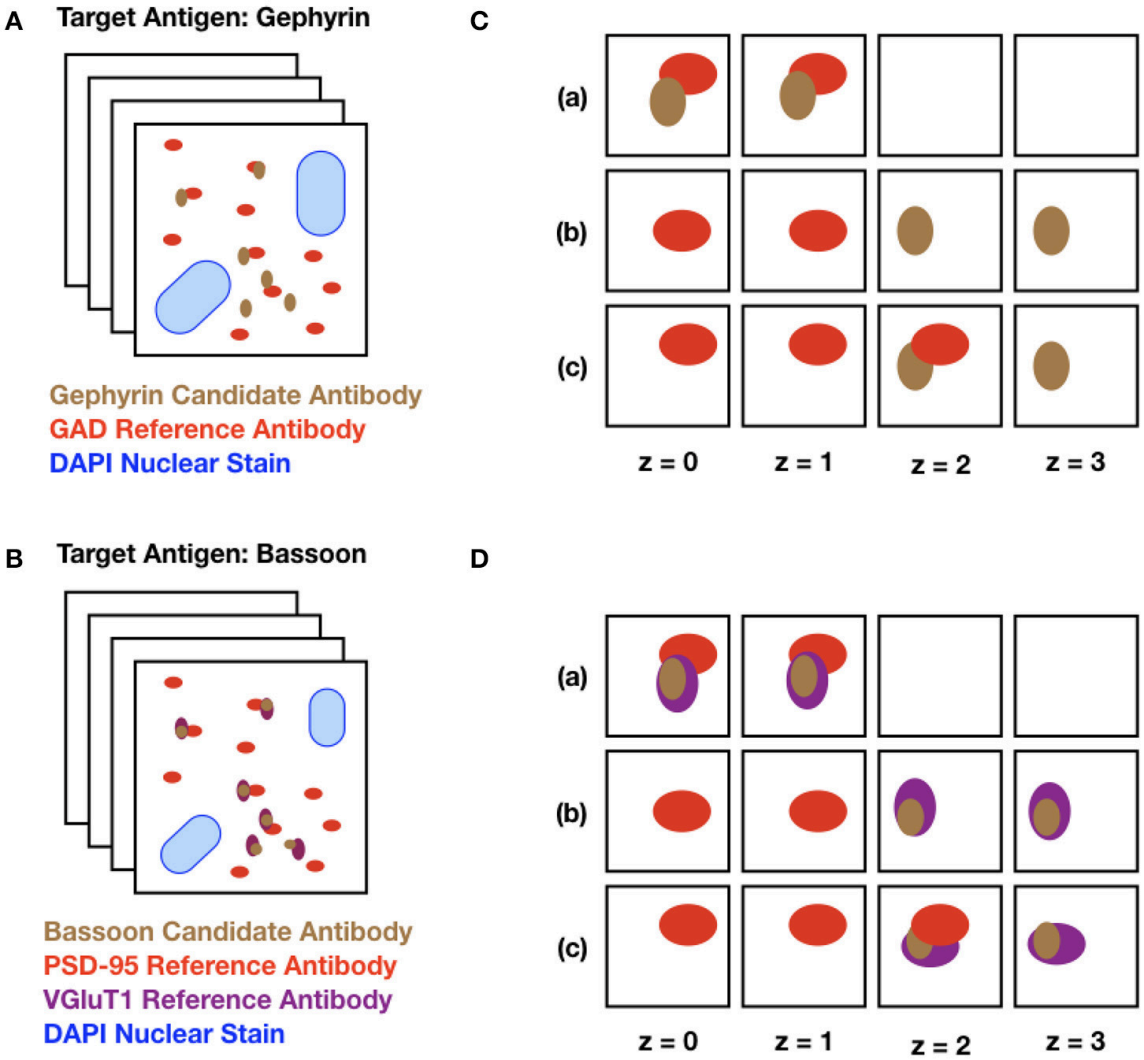
Schematic diagram of input datasets. **(A)** The target protein, gephyrin, is a postsynaptic protein at inhibitory synapses, expected to be adjacent to GAD, a presynaptic protein abundant at inhibitory synapses. The set of squares represents a stack of images from serial ultrathin sections from mouse neocortex, double labeled with a gephyrin candidate antibody (brown dots) and a previously validated antibody against GAD (red dots). The large blue blobs represent DAPI, a marker for cell nuclei. **(B)** Identical setup, but with Bassoon (a presynaptic protein present in excitatory synapses) as the target protein. In this example, the tissue is labeled with two reference antibodies to excitatory synapses, the presynaptic protein VGluT1 (purple dots) and the postsynaptic protein PSD-95 (red dots). **(C)** Different combinations of puncta are detected on sections (*z* = 0 to *z* = 3) through a synapse. The candidate and the reference antibody can be present alongside each other on the same section (a), they can lie adjacent in the z-direction (b), or they can be adjacent both in the same section and across multiple sections (c). **(D)** Identical setup as C with two reference antibodies depicted.

The data used for validating SACT were derived from serial sections of plastic-embedded tissue that was immunofluorescently labeled with a candidate antibody alongside one or more reference antibodies, chosen depending on the antigen. “Candidate antibody” refers to the antibody whose performance is being evaluated, and “Reference antibody” refers to an antibody previously validated for AT that marks a synaptic protein expected to colocalize with or be adjacent to the target of the candidate antibody. The colocalization or adjacency of these two (or more) markers indicates the presence of a target synapse with high probability. The selected area was imaged on at least 3 consecutive sections, the images were aligned into stacks (Figure 3), and the performance of the tested antibody was assessed using SACT.

Importantly, SACT is applicable to a variety of synaptic antigens with very different distributions, because the user defines the expected molecular composition and size of synapses where the antigen is present. Furthermore, the algorithm can be applied to new datasets without creating extensive manual annotations for each synapse subtype, unlike traditional classifiers such as support vector machines and deep learning used by other synapse detection algorithms (Busse and Smith, 2013; Kreshuk et al., 2014; Collman et al., 2015; Bass et al., 2017; Fantuzzo et al., 2017).

### 2.2. Punctum detection

Immunolabeling for synaptic proteins appears as small blobs or “puncta,” typically less than 1 μm diameter. Because synaptic structures are generally larger than the typical thickness of the individual sections used in our datasets (70 nm), the puncta corresponding to proteins that are abundant throughout the presynaptic or postsynaptic side span several sections and thus form three-dimensional puncta.

**Figure 4:**
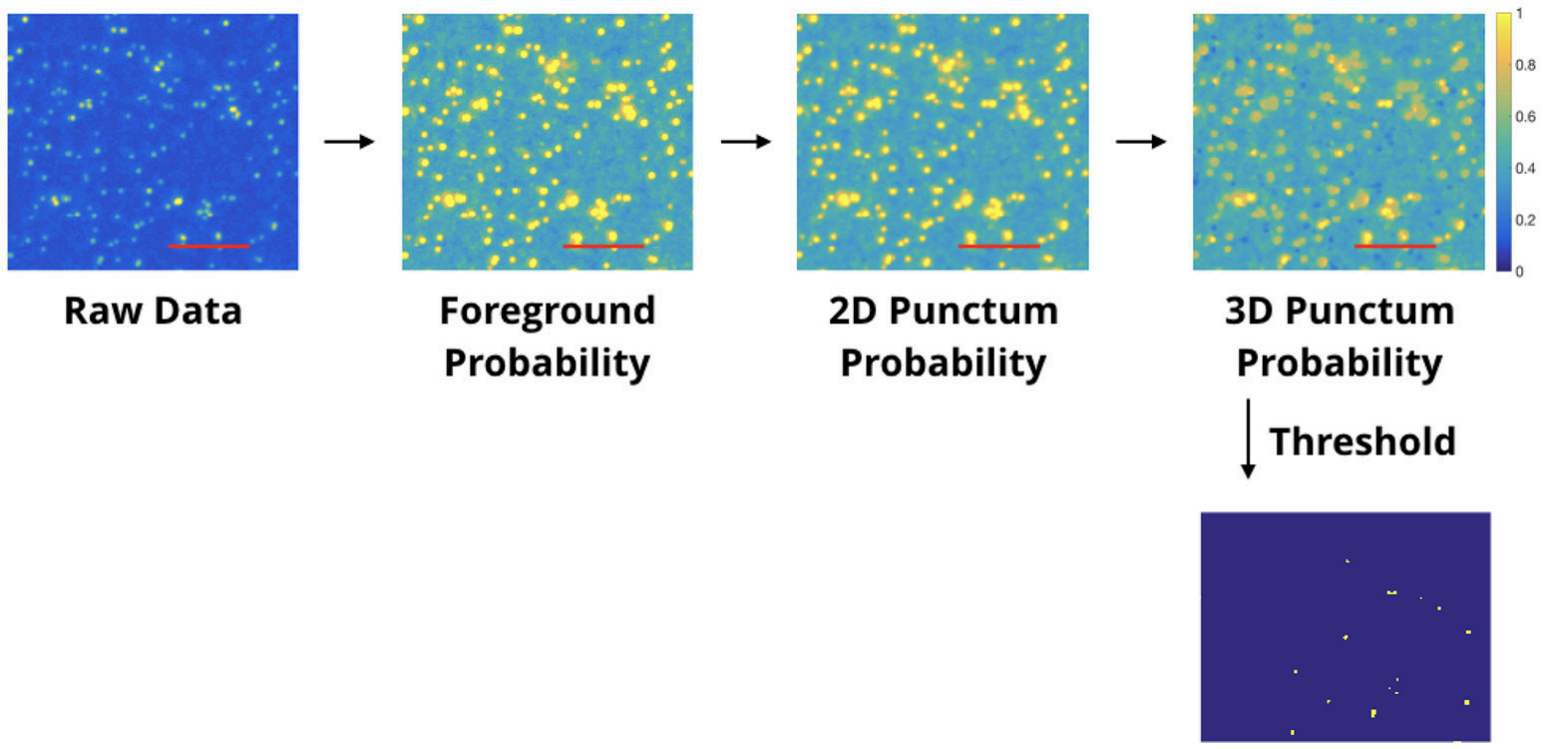
Automated punctum detection pipeline. This example illustrates the pipeline for antibody characterization. Each processed image shows the blobs/regions which have met criteria for being a punctum, and each successive panel adds a new requirement; the number of blobs considered as puncta decreases accordingly. In the final thresholded image, the blobs shown have met the requirement of spanning 3 slices and are centered on the slice shown. Other blobs which may appear “missing” are centered either on the slice before or after. The first box shows raw single-label immunofluorescence from a single slice. The second box is the output of a “foreground probability” step; the intensity value of each pixel encodes the probability it belongs to the foreground. The third box is the output of a “2D Punctum Probability” step (each pixel coding the probability that it belongs to a 2D blob). Pixels in the 4th box display the probability that a voxel belongs to a blob which spans a minimum number of slices. The final thresholded image is shown below. The threshold is established by visual observation; for this work, the threshold was set to 0.9 for the entire project. Red scale bars represent 5 μm.

The punctum detection method (Figure 4) is a special case of the synapse detection method and is adapted from it. It provides a way to take the input raw IF images from the microscope and output segmented 3D puncta, without having to set a threshold unique to every imaging session. The input is the volumetric image data and a user-defined query which includes the minimum expected 3D punctum size. Requiring a minimum 3D size minimizes the impact of random specks of noise generated during the image acquisition process and ensures that the immunolabeling is appropriately expressed across slices. For instance, a target protein that is abundantly expressed at a synapse (e.g., synapsin) should be detected across multiple slices at the current working resolution. Therefore, the presence of a punctum in only one slice likely indicates random noise, nonspecific labeling or fluorescent contaminant. On the other hand, there is little reason to assume that less abundant target proteins or those present at isolated nanodomains within synapses (e.g., many receptors or ion channels) need to span multiple slices through a synapse.

The probabilistic punctum detection algorithm involves three main steps. The first step transforms the data from the input raw IF images to a probability space. To do so, we create a Gaussian model for the background noise (other distribution models can be used as well) by assuming the entire input channel to be noise and the signal itself to be an outlier. For the Gaussian model, we use the input data's mean intensity value and standard deviation of the intensity values. The probability of a pixel belonging to the foreground is one minus the probability of belonging to the background. Next, we compute the probability that each pixel belongs to a 2D punctum. To do so, we multiply the probability values in a region defined by the user—the minimum expected 2D blob size. Usually, this is 0.2 × 0.2μ*m*, which corresponds to two pixels by two pixels. Then, we see if these 2D puncta exist in consecutive z slices. The number of expected slices is part of the user-defined minimum punctum size and is usually 0.14μ*m*, which corresponds to two slices. This minimum punctum size criteria reduces the effect of random noise. The output of this third step is a probability map, where the values at each pixel are the probability it belongs to a 3D punctum.

This probability map is thresholded to segment out 3D puncta detections. The threshold is established by manual observation (calibrated, if needed, with a small region of the data). The threshold was set to 0.9 for all the datasets in this project. Figure 4 shows the output of each step in this punctum detection pipeline. The initial panel shows a random slice / region of IF data. Each progressive panel shows a new requirement added, thus the number of “puncta” decrease accordingly. The last panel in Figure 4 shows the blobs/regions that have met the criteria necessary for being a punctum; not every blob shown in the first panel meets those requirements.

### 2.3. Synapse detection

Characterizing synaptic antibodies for AT immunolabeling of brain sections requires detecting synapses. Over the past few years, several synapse detection methods have been presented that use traditional machine learning paradigms for detection (Busse and Smith, 2013; Kreshuk et al., 2014; Collman et al., 2015; Bass et al., 2017; Fantuzzo et al., 2017). While they perform well, each requires the user to supply manually-labeled synapse annotations for training—an often impractical and tedious requirement for antibody validation, for which manual annotations would have to be created for each antibody. The probabilistic synapse detection method introduced in Simhal et al. (2017) does not require training data for synapse detection, making it an ideal synapse detector to use for antibody characterization. This approach extends the probabilistic puncta detection method to look for colocalization or adjacency between puncta from different synaptic proteins. For colocalization, the method looks for signal in a two by two pixel window. For adjacency, the method looks for blobs in a six by six pixel window. The algorithm takes as input the immunofluorscence data from the candidate and reference antibodies and the expected target synapse size (together referred to as the “query”) and outputs a probability map, where the value of each voxel represents the probability that it belongs to a synapse. This output can be thresholded to obtain the putative 3D synapse detections (see Simhal et al., 2017 for a detailed discussion). This algorithm is very flexible; as detailed below, the queries can be adapted to different data characteristics and analysis goals, further rendering it appropriate for antibody validation.

Generally, at least two known synaptic markers are required to unequivocally detect a synapse; therefore, some of the datasets contain two reference antibodies for synapse detection (Figures 3B,D). However, when screening multiple candidate antibodies, it is important to minimize the time and cost of the screen. When performed with caution using appropriate antibody combinations, even a single pre-validated antibody can generate interpretable synapse-specific data. For example, if the target protein for which an antibody is being evaluated is localized at the postsynaptic sites of inhibitory synapses, as is the case with gephyrin (Sheng and Kim, 2011), a reasonable strategy would be to label the tissue with an antibody against glutamic acid decarboxylase (GAD), a protein known to be specific to presynaptic terminals of GABAergic inhibitory synapses for which well-validated antibodies have already been identified. The corresponding query would then be to look for synapses containing immunolabeling for gephyrin and GAD (Figures 3A,C). If the target protein of interest is instead localized to excitatory synapses, the query may include proteins specific to excitatory synapses such as the postsynaptic protein PSD-95 (postsynaptic density 95) or the presynaptic protein VGluT1 (vesicular glutamate transporter 1). This flexibility makes this framework ideal for antibody characterization.

### 2.4. SACT measurements

In order to evaluate an antibody, a series of measurements are computed; each measurement captures an aspect of the antibody's performance that would be sought by an expert observer when manually interacting with the data. Each of these measurements provides unbiased quantitative information to help evaluate the intensity, specificity and sensitivity of immunolabeling obtained with a given antibody. These include the density of puncta, and the average punctum volume and standard deviation for each antibody, as well as the target synapse density (number of detected target synapses belonging to the specified subclass per volume), and target specificity ratio (the ratio of detected synapses to detected candidate antibody puncta). These measures provide the user a useful quantitative assessment of the data.

#### 2.4.1. Detected density of puncta

The density of the 3D puncta detected reflects the biological properties of the tissue (i.e., the abundance and distribution of the target protein in the tissue studied), as well as the intensity and specificity of the immunolabeling with a given antibody at the concentration used. If the labeling is unexpectedly sparse, it suggests that the antibody is insensitive or too highly diluted. If it is unexpectedly dense, it may indicate that the antibody is nonspecific or binds to many non-synaptic sites such mitochondria or other “sticky” subcellular sites. To determine the number of puncta detected in each channel, probability maps, where the value at every voxel is the probability it belongs to a 3D punctum, are computed as described in the previous section, and then thresholded^1^. The process is outlined in Figure 4. A density value of 0 indicates no puncta were found. The units (in this work) are in puncta per cubic micrometer. The 3D punctum density is then calculated:

$$\begin{array}{l} 3\text{D Punctum Density}=\frac{\text{Number of Detected }3\text{D Puncta}}{\text{Total Volume}} \end{array}$$

#### 2.4.2. Average punctum volume

**Figure 5:**
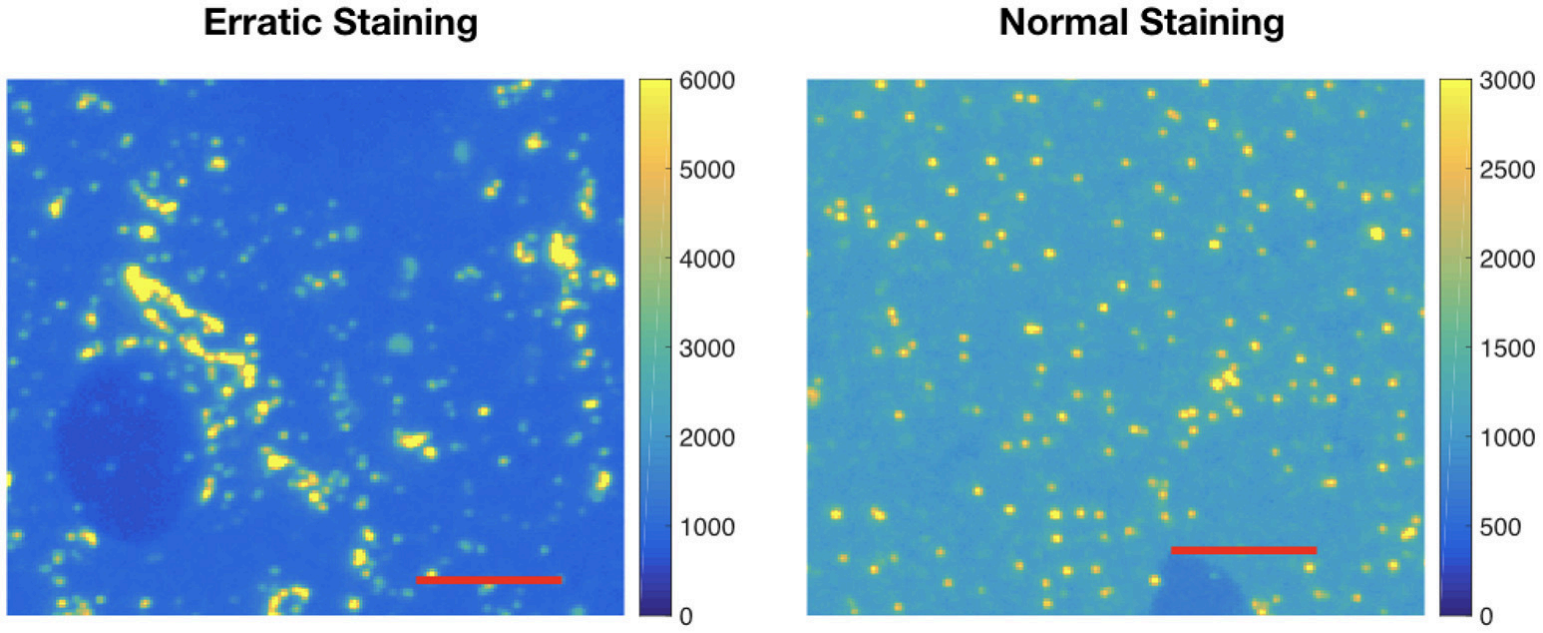
Example of erratic labeling. **Left:** Immunolabeling for collybistin (associated with GABAergic synapses) on a raw IF slice; note the very broad distribution of sizes for puncta. Clusters of immunofluorescent label are detected as one large punctum; for antibodies that give such labeling pattern, the average punctum size will be large and with a large size variance. **Right:** Relatively “normal” pattern of immunolabeling on a raw IF slice, using a different collybistin candidate antibody. This difference is automatically quantified by computing the average three dimensional punctum size and size variance. The image on the left has an average punctum size of 124 pixels and standard deviation of 1,350. The image of the right has an average punctum size of 10 pixels and standard deviation of 89 pixels. Each red scale bar is 5 μm.

After segmenting the immunolabeling data, the average volume of puncta and the standard deviation of the punctum volume are calculated. If the average punctum size is smaller or larger than expected, it may indicate a lack of efficacy or specificity for immunolabeling with that antibody. If the standard deviation of punctum volume is large, it may indicate erratic labeling. Either way, it is important to quantify the size distribution when comparing multiple candidate antibodies for the same target. Figure 5 shows an example of an antibody displaying a very large standard deviation, making it unlikely that the candidate antibody on the left will serve as a satisfactory marker for inhibitory (collybistin-positive) synapses. We could compute fuzzy volumes if we prefer to work with the probability maps instead of the thresholded data. In this work, the values are in pixels.

#### 2.4.3. Target synapse density

When evaluating a synaptic antibody, it is essential to confirm that the immunolabeling localizes at the expected population of target synapses. Target synapses are operationally defined by the relationship between two or more synaptic protein markers. This definition includes the size (in three dimensions) of each individual synaptic marker and a defined relationship (adjacent or colocalized) between the markers. Thus, the proposed SACT incorporates a probabilistic synapse detector, the thresholded output of which is the number of synapses detected with the candidate antibody. The target synapse density of a given volume is computed as

$$\begin{array}{l} \text{Target Synapse Density}=\frac{\text{Number of Detected Synapses}}{\text{Total Volume}} \end{array}$$

This measure is useful for evaluating antibodies against targets with a known distribution at synapses, where the density of synapses containing the target protein can be estimated. For example, ubiquitous markers of inhibitory synapses like gephyrin should be present at the large majority of inhibitory synapses, and should therefore have a synaptic density in rodent neocortex on the order of 0.15 synapses per μ*m*^3^ (Knott et al., 2002). A computed target synapse density substantially lower than expected may indicate low sensitivity of the antibody and/or insufficient concentration, while a target synapse density considerably above that expected likely reflects nonspecific (off-target) binding of the antibody. In this work, the units used are synapses per cubic micrometer. Note, the data is not thresholded until after searching for adjacency between puncta from different channels. The threshold for segmenting the resulting probability maps was set to 0.9 and was held constant for every experiment in the paper.

#### 2.4.4. Target specificity ratio

The target specificity ratio (TSR) represents the fraction of immunofluorescent puncta of the candidate antibody that are associated with a target synapse, detected as explained above. TSR is computed as

$$\begin{array}{l} \text{TSR}=\frac{\text{Number of Detected Synapses}}{\text{Number of Detected }3\text{D Puncta}} \end{array}$$

Thus, the target specificity ratio is a measure of how many times the candidate antibody being evaluated colocalizes with or is adjacent to the reference antibody compared to how many times it does not colocalize or lie adjacent with the reference antibody. TSR values range from 1 (every punctum detected has an associated detected synapse) to 0 (no detected punctum has an associated detected synapse); the higher the TSR, the lower is the magnitude of the nonsynaptic labeling obtained with that candidate antibody. Interpretation of this measurement will depend on the specific target; some synaptic proteins are almost exclusively present at synapses (e.g., synapsin), while others are also found at extrasynaptic locations (e.g., glutamate receptors). Therefore, the TSR reflects both the biological distribution of the target protein, and non-specific binding of the candidate antibody, but when comparing two antibodies against the same antigen, differences in TSR reflect differences in their specificity.

## 3. Materials

### 3.1. Datasets

The datasets presented here were created from adult mouse neocortical tissue that was prepared, immunolabeled, and imaged using standard methods of AT (Micheva et al., 2010; Collman et al., 2015). We used adult (3 to 4 months old) C57Bl/6J mice of both sexes for these experiments. Briefly, the tissue was chemically fixed using 4% paraformaldehyde in PBS, embedded in LR White resin, and cut into serial ultrathin sections (70 nm) that were mounted onto coverslips. One of the datasets from the automated ranking of the candidate monoclonal antibodies experiments (IRSp53) used tissue prepared in a different way: chemical fixation with 2% paraformaldehyde and 2% gluaraldehyde in PBS, followed by freeze-substitution and embedding in Lowicryl HM20 (Collman et al., 2015). The sections were labeled with indirect immunofluorescence using Alexa conjugated secondary antibodies (highly cross-adsorbed goat secondary antibodies against the relevant species, conjugated to Alexa 488, 594 or 647). Only Alexa 488 and 594 were used for the candidate primary antibodies. The difference in the theoretical lateral resolution of these two secondary antibodies calculated using Abbe's equation is 33 nm, which has little influence on our analysis for which the search area for colocalization is a 200 nm square. The samples were imaged on an automated wide-field fluorescence microscope (Zeiss AxioImager Z1, Zeiss, Oberkochen, Germany) with a 63x Plan-Apochromat 1.4 NA oil objective. The resulting images are not affected by many of the commonly occurring optical aberrations inherent to other immunofluorescence methods, because array tomography imaged sections are only 70 nm thick and the high-NA objective is always used at its design condition at the immediate contact of specimen and coverslip. While the exact size of the datasets varies, their general structure is consistent. Each dataset is composed of multichannel stacks of images from serial sections with (for the current data acquisition protocol) 100 × 100 nm pixel size and 70 nm slice thickness.

### 3.2. Primary antibodies

The antibodies used for the experiments presented here are listed in Table 1. Some of the antibodies used were tested in conjunction with screening of monoclonal antibody projects at the UC Davis/NIH NeuroMab Facility, consistent with our goal to facilitate testing of large panels of candidate antibodies in an efficient and objective fashion.

**Table 1:** Antibodies used in this study.

**Target protein**	**Host**	**Antibody source**	RRID^*^
Bassoon	Mouse	NeuroMab L124 project	RRID:AB_2716712
Cav3.1	Mouse	NeuroMab 75-206	RRID:AB_2069421
Cav3.1	Rabbit	Synaptic Systems 152 503	RRID:AB_2619850
Collybistin	Mouse	NeuroMab L120 project	RRID:AB_2650452
GAD2	Rabbit	Cell Signaling 5843	RRID:AB_10835855
Gephyrin BD	Mouse	BD Biosciences 612632	RRID:AB_399669
Gephyrin	Mouse	NeuroMab L106 project	RRID:AB_2617120 RRID:AB_617121 RRID:AB_2632414
GluA1	Rabbit	Millipore AB1504	RRID:AB_2113602
GluA2	Mouse	Millipore MAB397	RRID:AB_2113875
GluA3	Rabbit	Abcam ab40845	RRID:AB_776310
Homer1	Mouse	NeuroMab L113 project	RRID:AB_2629418
IRSp53	Mouse	NeuroMab L117 project	RRID:AB_2619741
GluN1	Mouse	Millipore MAB363	RRID:AB_94946
PSD95	Mouse	NeuroMab 75-028	RRID:AB_2292909
PSD95	Rabbit	Cell Signaling 3450	RRID:AB_2292883
Synapsin	Guinea pig	Synaptic Systems 106 004	RRID:AB_1106784
Synapsin	Rabbit	Cell Signaling 5297	RRID:AB_2616578
VGAT	Mouse	NeuroMab L118 project	RRID:AB_2650550
VGluT1	Guinea pig	Millipore AB5905	RRID:AB_2301751
VGluT1	Mouse	NeuroMab 75-066	RRID:AB_2187693

* *RRID: Research Resource Identifier. For the NeuroMab projects, the RRID of the antibody finally selected is listed; this selection was based on other factors in addition to the antibody performance evaluated using the current method*.

### 3.3. Computational analysis

The code and data used in this paper can be found at: https://aksimhal.github.io/SynapseAnalysis/. The website contains instructions on how to install and use the Synaptic Antibody Characterization Tool, alongside an example dataset a user can run right out of the box.

## 4. Results

### 4.1. Framework evaluation

The proposed synaptic antibody characterization and screening framework was evaluated via three different tasks. Each task demonstrates an aspect of the framework necessary for validating synaptic antibodies.

1. **Pairwise comparisons**. Comparing the performance of two previously validated antibodies against the same synaptic reference protein.
2. **Concentration comparisons**. Comparing the performance of a single antibody at different concentrations.
3. **Evaluating candidate monoclonal antibodies**. Comparing the performance of multiple candidate antibodies against the same synaptic target protein.

The first and second task validate the measurements proposed in the framework and involve only antibodies previously validated for array tomography. The third task evaluates the efficacy in a “real world” application—characterizing multiple antibody candidates whose suitability for AT has not yet been determined, and whose concentration is not known.

### 4.2. Pairwise comparisons

**Figure 6:**
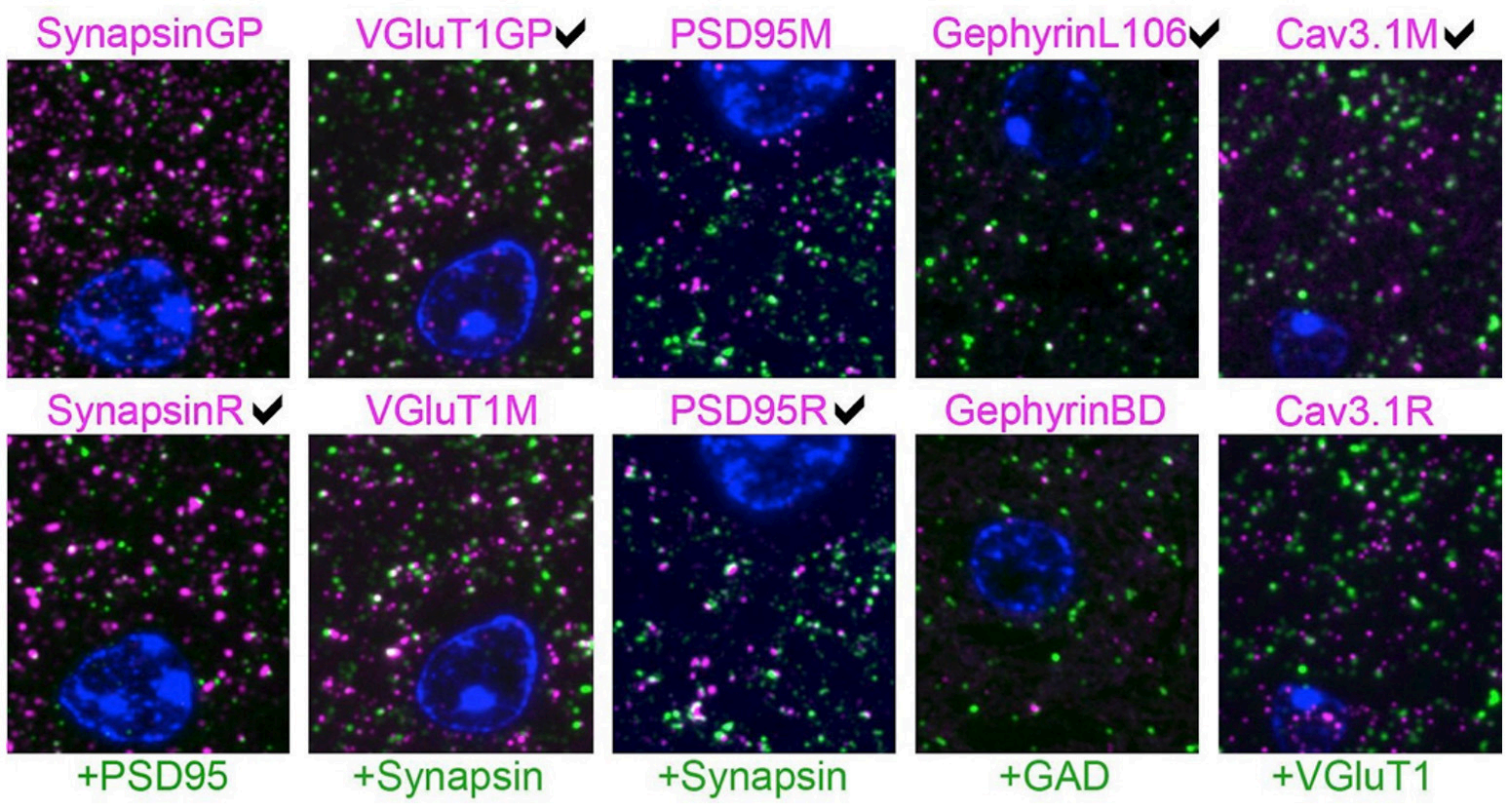
Pairwise comparison of immunofluorescence on single sections from mouse brain. Each column represents an experiment where two antibodies against the same target protein (magenta) were evaluated by double labeling with a reference antibody (green). The expert's visually-based preference is marked for each column. The sections are also labeled with the nuclear stain DAPI (blue). For each experiment, the two images are from the same section, except for the gephyrin results, where immunolabeling with the two gephyrin antibodies was performed on different sections. The SACT measurements for these images are shown in Table 2. Each image is 16 × 18 μm.

When two effective antibodies are available for use in a specific application, a common question is “which one is better?” To answer this question, we created five AT datasets, each with two previously validated antibodies used at concentrations previously determined to yield optimal immunolabeling. These antibody pairs were evaluated alongside an antibody for a different synaptic target protein, thoroughly validated for AT in prior studies (Micheva et al., 2010; Weiler et al., 2014). An example slice of each dataset is shown in Figure 6. The higher-scoring antibody was judged to be the one that had more puncta associated with the reference antibody (i.e., labels more synapses, true positives) and/or fewer puncta not associated with the reference antibody (false positives). In a dataset comparing different PSD-95 antibodies, the PSD95R antibody had more puncta adjacent to synapsin, without noticeably more synapsin-unrelated puncta, and was therefore evaluated as better performing than PSD95M. Cav3.1R had more puncta that are not adjacent to VGluT1, and also displayed nonspecific labeling of the cell nucleus, and was therefore judged to perform worse than Cav3.1M. In the VGluT1 dataset, differences between the two antibodies were more subtle, as shown by the measurements of target synaptic density and target specificity ratio (see Table 2).

**Table 2:** Results from pairwise antibody comparisons.

**Target protein**	**Candidate antibodies**	**Reference antibody**	**Punctum density (μm^3^)**	**Average punctum size / standard deviation (pixels)**	**Target synapse density (μm^3^)**	**Target specificity ratio**
Synapsin	Synapsin GP	PSD-95	2.01	15.9 */* 59.4	0.55	0.275
	**Synapsin R**		1.09	27.0 / 48.6	0.5	0.457
VGluT1	**VGluT1GP**	Synapsin	1.54	12.1 */* 30.2	0.44	0.283
	VGluT1M		1.27	11.0 */* 22.4	0.33	0.257
PSD-95	PSD-95M	Synapsin	0.95	10.7 / 101.7	0.54	0.568
	**PSD-95R**		1.14	25.4 / 39.9	0.79	0.691
Gephyrin	**GephyrinL106**	GAD	0.72	12.5 / 94.1	0.13	0.181
	GephyrinBD		0.46	17.9 / 263.1	0.08	0.175
Cav 3.1	**Cav3.1M**	VGluT1	0.58	7.4 / 14.0	0.33	0.568
	Cav3.1R		1.22	8.6 / 52.3	0.33	0.271

*Punctum density, average punctum size, and standard deviation of punctum size were all computed for each antibody tested. The names in bold in the second column represent the antibodies preferred by an expert based on visual examination. In each case, the measures automatically computed by the framework agree with the expert observer's judgement*.

For each dataset, the minimum expected marker size was set at 0.2 × 0.2 × 0.14μ*m*, corresponding to 2 pixels by 2 pixels by 2 slices. Each dataset was also independently evaluated and ranked by an expert observer (KDM) blind to the automatically computed results, based on visual examination of the immunolabeling. Two measures, target synapse density and target specificity ratio, were used to rank the two candidate antibodies (Table 2). When the antibodies are used at their optimal concentration, a higher measured target synapse density implies higher sensitivity of the antibody (since it detects the target protein at more synapses). A higher target specificity ratio (i.e., a higher proportion of detected immunolabeled puncta that are associated with synapses) indicates higher selectivity of the antibody for the protein of interest. The expert-preferred VGluT1 and PSD-95 antibodies scored higher on both sensitivity (target synapse density) and specificity (TSR), while others scored higher on only one of these measures. For both Cav3.1 and synapsin, the higher-scoring antibodies had higher TSR but gave target synapse densities comparable to the other antibody. In the case of gephyrin, both antibodies had similar TSR, but the expert-picked antibody had a higher target synapse density. These results illustrate the importance of using complementary measurements for antibody evaluation. The proposed framework provides multiple objective computations, and the user can pick the most suitable one(s) for a given task.

**Figure 7:**
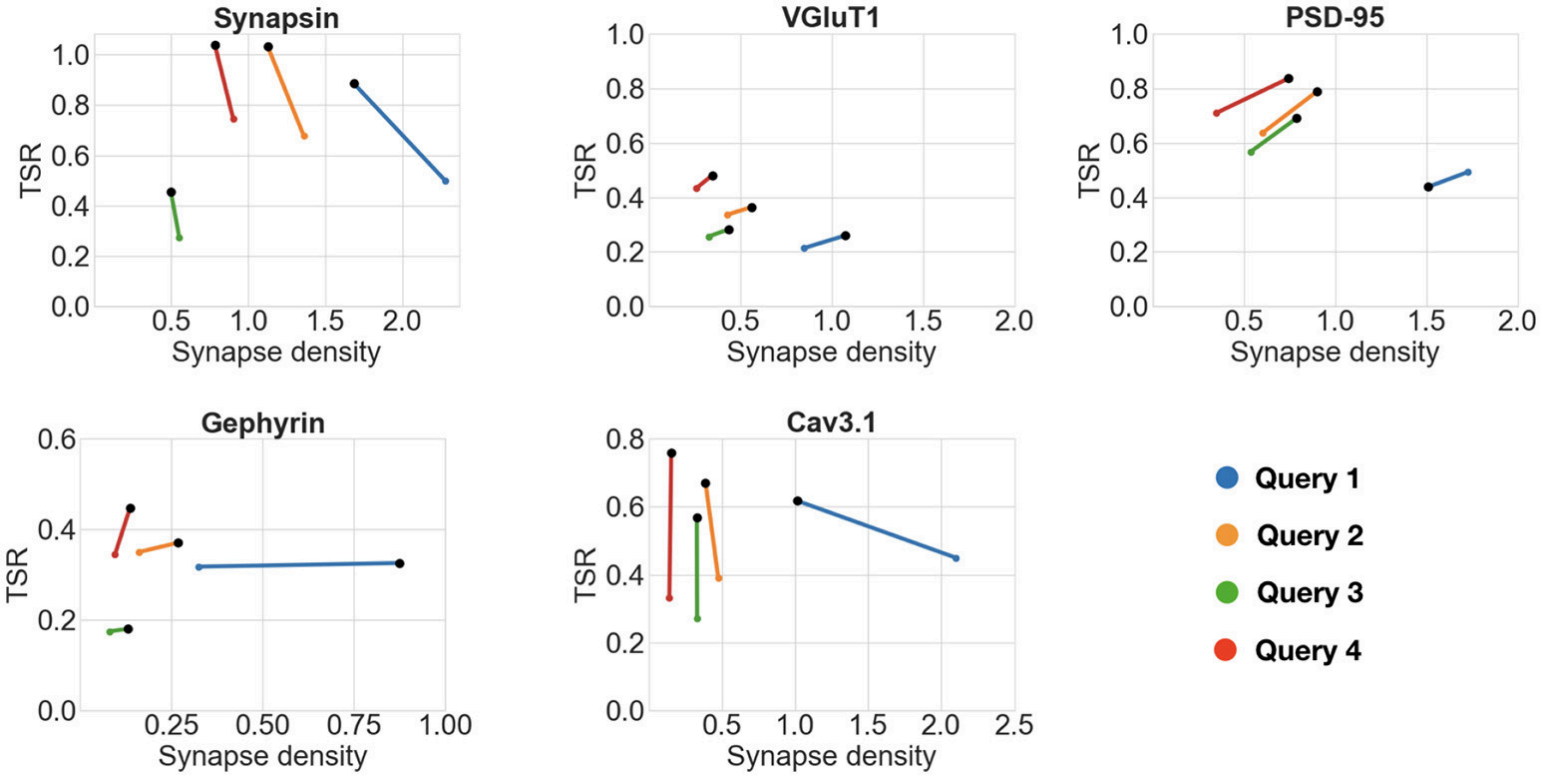
Impact of punctum size requirements on antibody comparisons. Black dots represent the higher scoring antibody. Each scatter plot shows the results of the comparison of two candidate antibodies against the same reference protein while varying the minimum synapse size requirements (queries numbered 1 through 4). The following are the minimum punctum size for the four queries. Query 1 (blue): candidate antibody—0.2 × 0.2 × 0.07μ*m*, reference antibody—0.2 × 0.2 × 0.07μ*m*. Query 2 (orange): candidate antibody—0.2 × 0.2 × 0.14μ*m*, reference antibody—0.2 × 0.2 × 0.07μ*m*. Query 3 (green): candidate antibody—0.2 × 0.2 × 0.14μ*m*, reference antibody—0.2 × 0.2 × 0.14μ*m*. Query 4 (red): candidate antibody—0.2 × 0.2 × 0.21μ*m*, reference antibody—0.2 × 0.2 × 0.07μ*m*. All queries gave consistent results for all five antibody pairs, except for Query 1 (see text for details). A TSR value of greater than one is an artifact of thresholding incorrectly splitting a punctum into two, it is remedied with simple morphological operations.

To evaluate the robustness of the framework, the same comparisons were performed using queries with smaller and larger minimum synapse size requirements (requiring puncta to span only one slice vs three slices), as shown in Figure 7. All queries gave consistent results for all five antibody pairs, except for Query 1, which defined a synapse as spanning only one slice; in two out of the five cases, Query 1 failed to unequivocally identify the otherwise highest scoring antibody. Thus, the use of even limited three-dimensional information from immunolabeling on serial sections enabled robust quantification of antibody performance.

This experiment illustrates the power and breadth of the proposed method. The queries can be designed by the user to take into account resolution, synapse type, and antibody binding target. Multiple queries can be run, and the antibody performance can be objectively evaluated with multiple measurements.

### 4.3. Concentration comparisons

The optimal concentration of an antibody, which is dependent on both its binding affinity for the target protein and the abundance of the target protein in the particular sample, must be determined experimentally. Too high a concentration of the antibody will lead to high background labeling (false positives), while too low a concentration will lead to sparse labeling (false negatives). The proposed framework quantifies the effects of antibody concentration on immunolabeling of AT sections, as evaluated by the target synapse density and target specificity ratio measures. As the antibody concentration decreases, the target synapse density is also expected to decrease.

**Figure 8:**
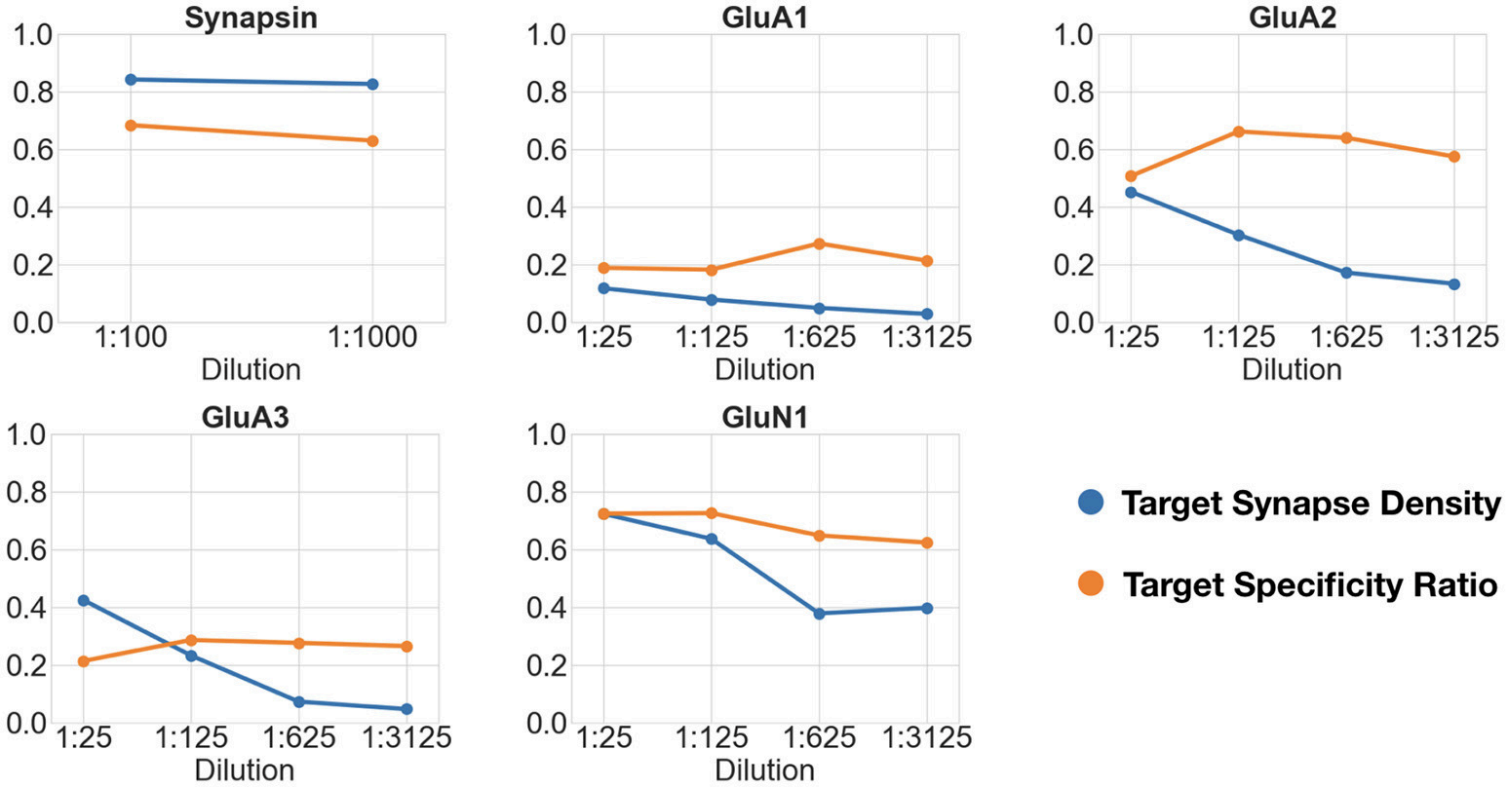
Changes in target synapse density (in synapses per cubic micrometers) and target specificity ratio as a function of antibody concentration. Each plot shows the target synapse density and target specificity ratio at different concentrations of the same antibody.

For this experiment, datasets were generated from a series of dilutions, as shown in Table 3 and Figure 8. For each dataset except GluN1 the minimum expected punctum size was 0.2 × 0.2 × 0.14μ*m*, corresponding to 2 pixels by 2 pixels by 2 slices. The minimum punctum size for GluN1 was (0.2 × 0.2 × 0.07μ*m*) due to inaccuracies in the alignment of this dataset that caused inconsistencies in the positions of synapses on adjacent slices (the proposed algorithm can be easily adapted to challenges in the data by changing the query).

**Table 3:** Five antibodies evaluated at different concentrations.

**Target protein**	**Candidate antibodies**	**Reference antibody**	**Punctum Density (μm^3^)**	**Average Punctum Size / standard deviation (pixels)**	**Target Synapse Density (μm^3^)**	**Target specificity ratio**
Synapsin	SynCS 1:100	VGluT1	1.23	41.6 / 133.6	0.84	0.68
	SynCS 1:1000		1.31	35.9 / 95.9	0.83	0.63
GluA1	GluR1 1:25	VGluT1	0.62	7.9 / 51.8	0.12	0.19
	GluR1 1:125		0.43	9.5 / 41.4	0.08	0.18
	GluR1 1:625		0.18	19.3 / 180.9	0.05	0.27
	GluR1 1:3125		0.13	38.7 / 1117.6	0.03	0.21
GluA2	GluR2 1:25	Synapsin	0.89	10.5 / 62.3	0.45	0.51
	GluR2 1:125		0.46	13.9 / 29.5	0.30	0.66
	GluR2 1:625		0.27	15.5 / 85.8	0.17	0.64
	GluR2 1:3125		0.23	15.9 / 103.8	0.13	0.57
GluA3	GluR3 1:25	VGluT1	1.99	6.5 / 11.8	0.42	0.21
	GluR3 1:125		0.81	8.4 / 16.4	0.23	0.29
	GluR3 1:625		0.26	10.8 / 23.5	0.07	0.28
	GluR3 1:3125		0.17	20.6 / 154.1	0.05	0.26
GluN1	GluN1 1:25	VGluT1	1	37.2 / 1541.0	0.72	0.72
	GluN1 1:125		0.88	17.8 / 208.2	0.63	0.72
	GluN1 1:625		0.58	22.0 / 748.9	0.37	0.64
	GluN1 1:3125		0.64	9.8 / 54.5	0.39	0.62

*In all experiments, a reference presynaptic antibody was included at its optimal concentration. The detected target synapse density decreases as the antibody concentration decreases. For GluN1, the background noise model changed to a Rayleigh distribution to better suit that specific dataset. All other datasets used the a Gaussian model for the background. See Simhal et al. (2017) for a detailed discussion*.

The first dataset tested the effect of a 10-fold change in concentration of an antibody against the general presynaptic marker synapsin, imaged in conjunction with immunolabeling for VGluT1, a presynaptic marker of excitatory synapses. The remaining datasets tested four sequential 5-fold concentration changes on immunolabeling with different glutamate receptor antibodies, and were evaluated against immunolabeling with antibodies for general presynaptic markers (synapsin) or markers of glutamatergic synapses (VGluT1). Synapsin, previously identified as a robust synaptic antibody for AT, performed equally well over the 10-fold concentration range as evaluated by both the target synapse density and target specificity ratio measurements. For each of the glutamate receptor antibodies, the measured target synapse density value decreased with increasing dilutions, as expected, while the target specificity value showed no consistent changes. Using the framework, we estimated that the optimal working range of the glutamate receptor antibodies tested lies within a dilution range of 1:25 to 1:125; further dilutions led to missing too many synapses without a substantial improvement in target specificity.

### 4.4. Automated ranking of candidate monoclonal antibodies

The generation of monoclonal antibodies begins with a high-throughput screening procedure that identifies numerous candidate antibodies, all of which must then be further investigated. Since only a small fraction of these candidate antibodies will exhibit robust and specific immunoreactivity in any given condition, it is important to screen as many candidate antibodies as possible for a given application. While some common antibody screens have been effectively automated (e.g., ELISA screens), screening on plastic sections from mammalian brain for antibodies that immunolabel specific populations of synapses must still be performed and analyzed manually by an expert observer, a difficult and labor-intensive process. Reasoning that the framework proposed here might facilitate the analysis of large-scale screens on tissue sections, we tested its performance by screening candidate monoclonal antibodies against synaptic target proteins generated at the UC Davis/NIH NeuroMab facility. This procedure is especially challenging because the concentration of antibody in hybridoma tissue culture supernatants is unknown, so immunolabeling must be performed at antibody concentrations that may differ for different candidate antibodies, and these concentrations may not be optimal.

Arrays from mouse neocortex were prepared using standard AT methods. For each dataset, we imaged sections immunolabeled with a set of candidate antibodies against the same target protein. For each dataset, at least two antibodies were applied: the candidate antibody raised against the target protein of interest and a validated reference antibody at its optimal concentration. The ranking of candidate antibodies was determined based on two measurements provided by the framework: target synapse density and the target specificity ratio. The target specificity ratio was the deciding factor in most cases. Target synapse density was used to exclude candidate antibodies with unreasonably high values based on previous biological knowledge: excitatory synapses are expected to have a density of ~1 per μ*m*^3^, and the inhibitory synapses a density of ~ 0.15 per μ*m*^3^ (Calverley and Jones, 1987; Schüz and Palm, 1989; Knott et al., 2002). Each dataset was blindly evaluated and ranked by an expert observer, based on visual examination of the images. Screening of the Bassoon candidate antibodies was performed in two rounds; the second round included only those candidates identified as best or unclear in the first round. These experiments addressed several questions: 1) Can the framework be used to correctly rank the performance of multiple candidate antibodies? 2) What is the minimum number of reference antibodies required to accomplish this? and 3) What is the optimal minimum punctum size needed? Six datasets, ranging from 4 to 19 different candidate antibodies each, were analyzed.

#### 4.4.1. Can the framework correctly rank the performance of multiple candidate antibodies?

**Figure 9:**
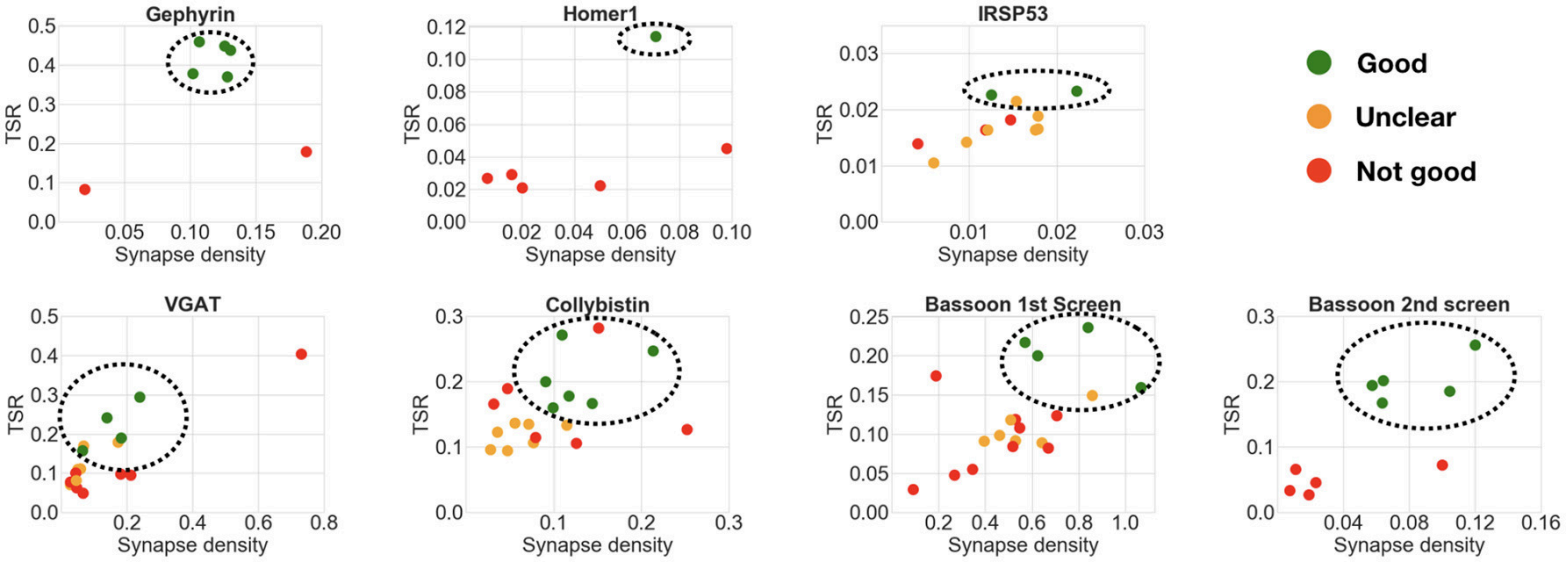
Comparison of multiple candidate antibodies. Each scatter plot shows the computed target synapse density and target specificity ratio of multiple candidate antibodies, with the best-ranking candidates circled. Expert ranking is color-coded: green—best, orange—unclear, red—fail. The outlier in the VGAT scatter plot was not included in the best candidate antibodies selection, because of the abnormally high synaptic density (0.7 per μ*m*^3^ compared to target max density of 0.15 per μ*m*^3^). Screening of the Bassoon project was performed in 2 rounds: the candidate antibodies identified as best or unclear in the first round were screened again with adjusted concentrations.

Analysis of the six datasets demonstrated an excellent correspondence between the framework's ranking and expert evaluation of candidate antibody performance. The results are summarized in Table 4 and Figure 9. The computed measures not only allow the relative ranking of antibody performance, but also give an indication of the absolute utility of an antibody. For example, in the case of IRSp53, the two expert-preferred antibodies were ranked higher by the SACT than the other antibodies. However, the SACT measurements indicated that overall none of the IRSp53 antibodies tested were performing sufficiently well, because the TSR was extremely low (<0.03).

**Table 4:** Summary of candidate antibody comparisons.

**Target protein**	**Reference antibodies**	**Total no. of candidate antibodies tested**	**No. of good candidates chosen by expert**	**No. of good candidates chosen by SACT**	**SACT false negatives**	**SACT false positives**
Gephyrin	GAD	7	5	4	1	0
Homer	PSD95	4	1	1	0	0
IRSP53	PSD-95, VGluT1	12	2	2	0	0 (+1 unclear)
VGAT	GAD	17	4	4	0	0 (+2 unclear)
Collybistin	GAD	19	6	6	0	1
Bassoon 1st exp.	Synapsin	19	4	4	0	0 (+1 unclear)
Bassoon 2nd exp.	VGluT1, PSD-95	10	5	5	0	0

*Multiple candidate antibodies against the target protein were ranked using measurements from the proposed SACT. The candidate antibodies were independently evaluated by visual inspection of the immunofluorescence images. For each target protein, the reference antibodies were chosen according to the known characteristics of synapses expressing the target protein. Without an objective and automated tool to quantify antibody performance, all antibody evaluations are subjective (as is the standard today). The framework proposed in this work is a first step toward having synaptic antibody quantification be a routine part of antibody evaluation*.

#### 4.4.2. What is the minimum number of reference antibodies?

**Figure 10:**
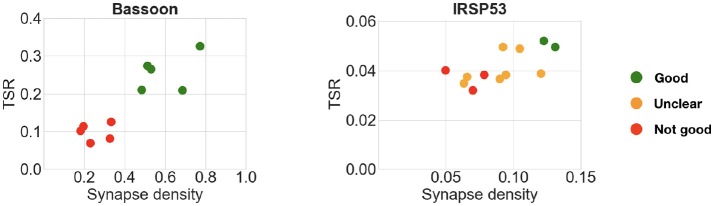
Comparison of multiple candidate antibodies using two reference synaptic antibodies. **Left:** Bassoon with PSD-95 and VGluT1. **Right:** IRSp53 with PSD-95 and VGluT1. Expert ranking is color coded: green—best, orange—unclear, red—fail. Compare with Figure 9.

The previous experiments with pairwise or concentration comparisons were performed using only one reference antibody. In those cases, the tested antibodies were already known to recognize their target in plastic sections; it is therefore reasonable to assume that the combination of one reference synaptic antibody with one tested synaptic antibody will generate synapse-specific data. To verify whether an additional reference antibody may offer an advantage when screening new antibodies, two of the datasets included both presynaptic and postsynaptic reference antibodies. In these two datasets, the performance of the query containing an additional reference antibody was compared to the standard single reference antibody query used in the previous experiments. In both cases, the results of the two queries were very similar (compare Figure 10 with Figure 9), suggesting that inclusion of a second reference synaptic antibody is unnecessary for the purpose of screening large sets of candidate antibodies.

#### 4.4.3. What is the optimal minimum punctum size?

**Figure 11:**
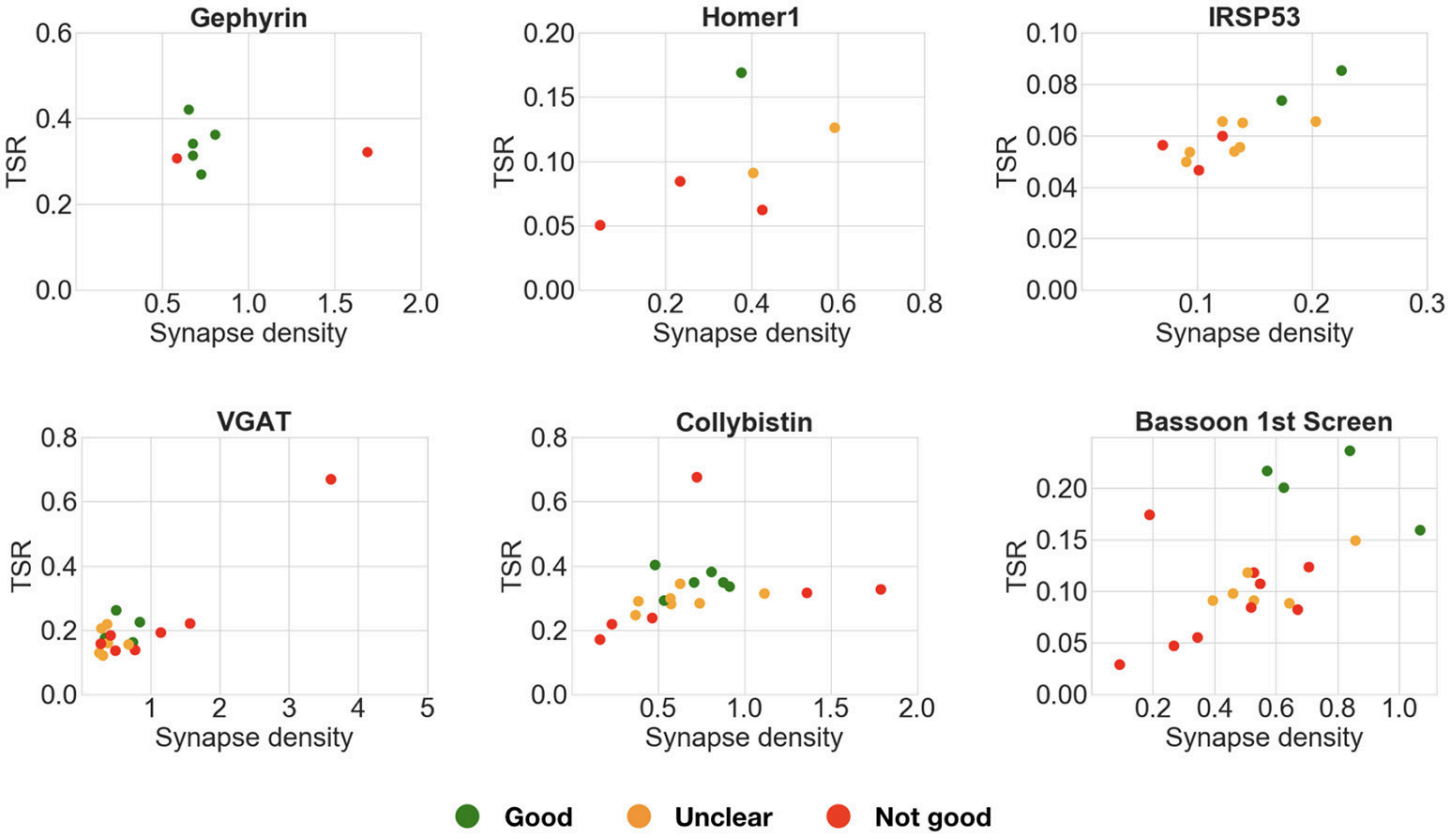
Comparison of multiple candidate antibodies. These plots compare multiple candidate antibodies using an alternative query requiring the puncta to be present on only 1 slice, instead of 2 as in Figure 9. Each scatter plot shows the target synapse density and target specificity ratio of multiple candidate antibodies against the same target protein. Expert ranking is color coded: green—best, orange—unclear, red—fail. In many of these cases (gephyrin, VGAT, collybistin), the more permissive 1-slice query does not allow correct selection of the best performing candidate antibodies.

The pairwise antibody comparison experiments showed that the results were not affected by the stringency of the query, except in cases when the minimum puncta requirements were too permissive (smallest synapse size: labels present on 1 slice). Therefore, to screen multiple candidate antibodies, we generally chose queries of medium stringency, requiring the labels to be present in two consecutive slices. This strategy worked very well for candidate antibodies directed against abundant synaptic proteins (gephyrin, Homer1, IRSp53, VGAT, collybistin). In contrast, the permissive query, which required the labels to be present on only one section, gave inconclusive results in most of these cases (Figure 11). The first round of screening for Bassoon antibodies was an exception, because it yielded clearer results with a one-section query. This is likely due to the wide variations in concentration of the candidate antibodies present in the tissue culture supernatants used for screening, many of which required subsequent dilution, as performed in the second round of testing. In this second round with adjusted concentrations, the two-section query performed well, as seen for the other abundant synaptic target proteins. These experiments suggest that it is best to start an antibody evaluation using a query that requires the labels to be present in two sections. The top-ranking antibodies based on such a query can then be selected and visually examined by experts to confirm their performance.

## 5. Discussion

The present report introduces an effective framework for automated characterization and screening of antibodies for AT. The framework provides a number of automatically computed characteristics, such as target synapse density and target specificity ratio, that reflect the sensitivity and specificity of immunolabeling with a given antibody. Taken together, these computed characteristics provide an objective way to characterize and compare the performance of different antibodies against the same target, simplifying the process for selecting antibodies best suited for AT. When evaluating multiple candidate antibodies, this represents an efficient method to identify a small number of promising antibodies for further evaluation, which includes assays in knockout mouse and other relevant controls (Gong et al., 2016).

The Synaptic Antibody Characterization Tool (SACT, the implementation of the framework) is designed to be a flexible tool for antibody screening. Because it is query-based, it allows the user to define the molecular composition and size of synapses expected to contain the antigen. This flexibility is advantageous for synaptic antibody screening because the query can be designed to focus on different synapse subtypes, depending on prior biological knowledge (e.g., what combinations of proteins are likely to be present, and where the antibody target is expected to be located). Its inherent flexibility should allow this approach to be used also to validate antibodies that target other subcellular structures, ranging from the nodes of Ranvier on myelinated axons, to mitochondria, to histone markers in the nucleus. The method works with a wide selection of reference antibodies, which need not colocalize with the tested antibody. For example, antibodies to gephyrin and collybistin, both postsynaptic proteins, were evaluated using the presynaptic marker GAD as reference. The flexibility in reference antibody selection enables users to optimize the use of their available antibody stocks. With further practical experience we anticipate that a restricted group of well-characterized antibodies will be adopted as controls for each target category.

Our experiments demonstrate that SACT provides a robust method for antibody screening, ranking antibodies based on quantitative measures of their performance. In the pairwise comparisons of antibodies, there was 100% agreement between the expert ranking and the automated antibody ranking based on target synapse density and target selectivity ratio. Variations in the size requirement did not affect the ranking, as long as synapse detection was based on more than one slice. Even when a synapse was required to be present on only one slice, performance was only modestly degraded, such that the outcome measures for some antibody pairs were ambiguous. The present approach accommodates variations in antibody concentration, as demonstrated by the experiments with multiple candidate antibodies from monoclonal antibody projects, which showed a high correlation between the ranking by algorithm and by expert evaluation of candidate antibody performance in all six datasets, even though the concentration of antibodies in the hybridoma tissue culture supernatants used for screening was unknown and intrinsically variable. This insensitivity to antibody concentration is very important in practice when evaluating multiple antibodies; by eliminating the need for immunolabeling with series of antibody dilutions, it substantially reduces the amount of work involved.

There are some limitations to the use of the proposed framework for antibody validation. This is not a stand-alone tool for generic antibody validation; it is designed to specifically address the performance of the antibody for immunofluorescence AT, and must be used along with other tests and controls. For example, SACT does not test for cross-reactivity with other proteins. A second limitation is that this approach requires prior knowledge of the expected distribution of the antigen (or some other characteristic to use for reference), especially if it is found only in a small population of synapses. In such cases it will be important to ensure that the tissue sample used for immunolabeling contains such synapses at a reasonable density and/or includes a reference marker to independently identify this population.

A number of technical issues can interfere with performance. Proper alignment of the sections in the imaged series is required to ensure that position of synapses is consistent on adjacent sections. In one of the concentration comparison experiments with GluN1, inaccuracies in the alignment led to poor performance of the algorithm when using the standard size requirement of a synapse to be present on at least two consecutive sections. In this case, a one-slice size requirement was successfully used, but we show that this approach will not always work. To fully benefit from the advantages of using three-dimensional information from multiple serial slices, one must ensure that the datasets are well aligned. Another technical issue to consider is possible bleed-through during the fluorescent imaging, which can cause the false impression of colocalization between the tested and reference antibodies.

When carefully planned and executed to avoid pitfalls, the automated framework described here can be used to identify and characterize antibodies against a wide assortment of synaptic target proteins that yield robust and specific immunolabeling in plastic sections of brain tissue. This is particularly important because the nature of synaptic processing is still poorly understood, and many basic questions remain. For example, how many different types of synapse exist? How do these different types vary over different brain areas? How does their distribution change over time? With experience? Under pathological conditions? For questions of this nature, it is important to objectively assess a large number of individual synapses, and a large number of different molecules at each synapse, as can be done using AT. Identifying reliable synaptic antibodies for AT will remove a major limitation for such studies and allow a better understanding of synapses.

## Data availability statement

The code and data used in this paper can be found here: https://aksimhal.github.io/SynapseAnalysis/.

## Ethics statement

All procedures related to the care and treatment of animals were approved by the Administrative Panel on Laboratory Animal Care at Stanford University.

## Author contributions

AS, RW, GS, and KM designed the study, with KM leading the effort. AS and GS designed the computational algorithm. AS wrote the code and ran the tests. KM provided the data and evaluation. BG and JT provided the tested antibodies. SS oversees the effort. AS, RW, GS, and KM wrote the core of the paper with contributions from all authors.

### Conflict of interest statement

SS and KM have founder's equity interests in Aratome, LLC (Menlo Park, CA), an enterprise that produces array tomography materials and services. SS and KM are also listed as inventors on two US patents regarding array tomography methods that have been issued to Stanford University. The remaining authors declare that the research was conducted in the absence of any commercial or financial relationships that could be construed as a potential conflict of interest.

## Footnotes

^1^The threshold could be avoided by changing the formula to be the sum of the probabilities instead of the thresholded number of 3D puncta. This applies to the other measurements described next as well.
